# Total Ischemic Time on In-Hospital Complication Predictor in ST-Elevation Myocardial Infarction (STEMI) Patients With Renal Dysfunction

**DOI:** 10.7759/cureus.33903

**Published:** 2023-01-17

**Authors:** Harutyun Petrosyan, Hamlet Hayrapetyan, Shahen Torozyan, Ksenia Plotnikova, Mukhammad Ashurov, Anastasiia Veprintseva, Valeriia Kimutsadze, Veronika Kimutsadze, Rebeka Hakobova, Norik Kazaryan

**Affiliations:** 1 Cardiovascular Surgery, Erebuni Medical Center, Yerevan, ARM; 2 Pain Management, Pain Clinic, Krasnodar, RUS; 3 Faculty of Medicine, First Moscow State Medical University, Moscow, RUS; 4 Faculty of Medicine, Peoples’ Friendship University of Russia, Moscow, RUS; 5 Surgery, Ust-ischim District Hospital, Omsk, RUS; 6 Surgery, Astghik Medical Center, Yerevan, ARM

**Keywords:** gfr, renal dysfunction, percutaneous coronary intervention, total ischemic time, stemi

## Abstract

Aim

The purpose of this study was to examine the impact of total ischemic time (TIT) on in-hospital complications in acute ST-elevation myocardial infarction (STEMI) patients with renal dysfunction (RD).

Methods

The study included a total of 116 patients. All patients underwent percutaneous coronary intervention. Glomerular filtration rate (GFR) was < 60 ml/min/1.73 m^2^ in all patients. The patients were split into two groups based on the TIT value. All eligible patients were assigned to two groups according to TIT: Group 1 comprised 54 patients with ≤ 6-hour TIT and Group 2 consisted of 62 patients with > 6-hour TIT. The groups' other characteristics were similar. The composite rate of pulmonary edema and cardiogenic shock were compared between groups.

Results

The mean TIT in Group 1 was 4.37 ± 1.35 and in Group 2 was 9.03 ± 1.59 (p < 0.0001). The incidence of pulmonary edema or cardiogenic shock was higher in Group 2 than in Group 1: 16.1% and 3.7%, respectively (p = 0.034).

Conclusion

STEMI patients with RD and higher TIT were more likely to develop pulmonary edema and cardiogenic shock.

## Introduction

Clinical trials have made tremendous progress in establishing the importance of gender and, more recently, the role of diabetes and obesity as key comorbidities for coronary artery disease [1,2]. Renal disease hasn't gotten the same kind of attention; in fact, cardiovascular events account for more than half of all deaths among people with end-stage renal disease [3,4]. The severity of renal insufficiency correlates to an increased risk of death from heart failure in several studies [5]. With deteriorating renal function comes an increased risk of cardiovascular disease [6-8]. Renal impairment at the time of admission in patients with acute myocardial infarction (AMI) should be a warning sign of a higher risk of hospital complications. According to current recommendations, patients with renal impairment should get the same care as other acute coronary syndrome patients, with the exception of prescription doses [9,10]. Those with renal dysfunction (RD) may benefit from an invasive procedure more than patients without RD in terms of absolute benefits; nevertheless, the chance of unfavorable results is also higher in RD patients [11,12]. It's crucial to figure out what's causing patients with renal impairment to have bad outcomes. The central treatment for acute ST-elevation myocardial infarction (STEMI) is myocardial reperfusion with primary percutaneous coronary intervention (PCI), which must be done quickly. As a result, the time from arrival at the emergency room to ballooning, known as door-to-balloon time (DTB), has gained a lot of traction among cardiologists and is currently regarded as the most critical quality measure for primary PCI [13]. Other delays in the STEMI timeline, such as the period between the start of chest discomfort and the arrival of a PCI-capable hospital, received less attention [13]. The total ischemic time (TIT), measured from symptom onset to the provision of reperfusion therapy, is so critical for the prognosis of STEMI patients, and it should be carefully considered when determining the time to reperfusion [14]. It may be a more powerful prognostic indicator than DTB time, as an increase in microvascular blockage area has been linked to extended ischemia [15]. Gao and co-authors found that in patients with STEMI with ejection fraction (EF) < 50%, TIT was independently correlated with all-cause mortality in the hospital after PCI [16]. The findings of this study also reveal that acute kidney injury, Killip, and thrombolysis in myocardial infarction (TIMI) grade are substantial risk factors for in-hospital death in STEMI patients [16]. At a one-year follow-up, another study found that persistent ischemia was associated with death, rehospitalization, and revascularization [17]. However, if the effect of TIT is confirmed in terms of long-term prognosis, the short-term prognosis outcomes are conflicting. Lubovich and co-authors, for example, found no statistically significant difference in 30-day mortality for STEMI patients based on TIT [18]. It was assumed that the combination of many risk factors in myocardial infarction would result in increased complications. As previously said, TIT is a predictive risk factor in STEMI patients, and the purpose of this study was to compare in-hospital complications in STEMI patients with RD based on TIT value.

## Materials and methods

Study design and population

This is a single-center, observational, prospective study. For this study, STEMI patients with RD were chosen and divided into two groups based on their TIT values. New evidence of STEMI on electrocardiography with an elevation of myocardial necrosis enzymes as high-sensitivity troponins, presence of typical angina or dyspnoea that could be interpreted as angina-equivalent, decreased glomerular filtration rate (GFR) at the time of admission, and emergency PCI were the inclusion criteria. Patients with STEMI who underwent only an urgent coronary angiography without PCI were not included in the research. RD was considered in patients who had GFR less than 60 ml/min/1.73 m^2^ at the time of admission․ All eligible patients were assigned to two groups according to TIT: Group 1 comprised 54 patients with ≤ 6-hour TIT and Group 2 consisted of 62 patients with > 6-hour TIT. The Modification of Diet in Renal Disease (MDRD) formula was used to calculate GFR, which takes creatinine, age, gender, and race into account [19]. The patients were all of the Caucasian race. During the first hours after admission, all patients' demographic, clinical, and angiographic data were collected, including age, gender, cardiovascular risk factors such as hypertension and diabetes, and prior myocardial infarction STEMI localization (Table 1). Iohexol was used as a contrast agent. Every factor, as well as the standards for diagnosing STEMI and acute heart failure (AHF), were defined in the European Society of Cardiology (ESC) guideline on STEMI management [20].

**Table 1:** Baseline demography and clinical characteristics stratified according to total ischemic time (TIT). Presented values are % or mean ± standard deviation TIT: total ischemic time, GFR: glomerular filtration rate, MI: myocardial infarction, STEMI: ST-elevation myocardial infarction, LV EF: left ventricular ejection fraction

Characteristics	Total n = 116	Group 1 n = 54	Group 2 n = 62	p-value
TIT	6.9 ± 2.7	4.3 ± 1.3	9.0 ± 1.6	< 0.0001
GFR (ml/min/1.73 m^2^)	48.2 ± 10.4	47.9 ± 10.2	48.4 ± 10.6	0.79
Age, years	66.2 ± 10.1	64.8 ± 10.9	67.4 ± 9.4	0.17
Male	75 (64.7%)	38 (70.4%)	37 (59.7%)	0.31
Prior MI	13 (11.2%)	5 (9.3%)	8 (12.9%)	0.53
Hypertension	79 (68.1%)	35 (64.8%)	44 (71%)	0.48
Diabetes	26 (22.4%)	12 (22.2%)	14 (22.5%)	0.96
Anterior STEMI	54 (46.6%)	21 (38.9%)	33 (53.2%)	0.18
Multivessel disease	54 (46.6%)	24 (44.4%)	30 (48.4%)	0.67
Killip > II class at the time of admission	4 (3.4%)	2 (3.7%)	2 (3.2%)	1.0
LV EF (%)	39.1 ± 8.5	39.9 ± 9.8	38.5 ± 7.25	0.36
Door to balloon time	1.0 ± 0.5	1.1 ± 0.4	1.0 ± 0.5	0.24

Data collection

Patients' information was gathered, including general information, vital signs, auxiliary examination results, chest pain-related information, and information on interventional therapy and medication. The age and sex of the participants were included in the general data. Hypertension, diabetes, and myocardial infarction were all mentioned in the past medical history. Killip classification was included in vital signs. Infarct localization and left ventricular ejection fraction (LV EF) were among the auxiliary examination results. TIT was included in the chest pain data.

Hemodynamic evaluation

Patients with AMI who had no signs or symptoms of heart failure were classified as Killip I; those with left heart failure were classified as Killip II if the moist rales of both lungs accounted for less than half of the lung fields; those with AMI complicated by pulmonary edema and small, large, moist and dry rales of the entire lung were classified as Killip III; and those with AMI complicated by cardiogenic shock were classified as Killip IV [21].

Treatment protocol

The study excluded patients with acute cerebrovascular disease, cancer, severe valvular heart disease, or other severe non-cardiac illnesses with a survival time of less than a year. Screening failed for six patients in the first, and three patients in the second group due to exclusion criteria. In addition, patients who declined to take part in the study were excluded (four patients for each group). Following the ESC guidelines on AMI in patients with ST-segment elevation, primary PCI as a standard procedure through the radial artery route was performed on all enrolled STEMI patients in two groups [20]. All of the patients were given as many stents as the infarct-related artery required clinically. All of the patients in both groups received standard pharmacological treatment for STEMI, which included heparin, clopidogrel, aspirin, statins, beta-blockers, diuretics, angiotensin-converting enzyme inhibitors, angiotensin II receptor blockers, and other medications, as per current guidelines (Table 2) [20].

**Table 2:** The groups were comparable in terms of received drugs. LMWHs: low molecular weight heparins, ACE: angiotensin-converting enzyme, ARBs: angiotensin II receptor blockers

Received drugs	Total n = 116	Group 1 n = 54	Group 2 n = 62	p-value
Aspirin	116 (100%)	54 (100%)	62 (100%)	–
Clopidogrel	116 (100%)	54 (100%)	62 (100%)	–
Heparin and LMWHs	116 (100%)	54 (100%)	62 (100%)	–
Statins	116 (100%)	54 (100%)	62 (100%)	–
B-blockers	93 (80.2%)	43 (79.6%)	50 (80.6%)	1.0
ACE inhibitors or ARBs	94 (81%)	43 (79.6%)	51 (82.3%)	0.81
Aldosterone antagonists	39 (33.6%)	18 (33.3%)	21 (33.8%)	0.95
Calcium antagonists	26 (22.4%)	11 (20.8%)	15 (24.2%)	0.66
Loop diuretics	45 (38.8%)	19 (35.1%)	26 (41.9%)	0.74

Study outcomes

The treating physician reported in-hospital complications such as pulmonary edema, cardiogenic shock, and in-hospital death (ID). Between groups, the incidences of in-hospital complications were compared at the end of their hospital stays.

Statistical analysis

Statistical Product and Service Solutions (SPSS) (IBM SPSS Statistics for Windows, Version 26.0, Armonk, NY) software was used to collect survey data and digitize it. The researcher used standard statistical methods. All calculated p values were two-tailed, and statistical significance was determined when < 0.05. The mean and standard deviation (SD) are used to summarize continuous variables, while frequency and group percentage are used to summarize categorical variables. The patient data in the study were analyzed using statistical student's t-test, Pearson's chi-square test, and Fisher’s z-test as necessary.

## Results

All 116 patients who had acute STEMI were thus included. In Group 1 were 64.8±10.9 years old on average, compared to 67.4±9.4 in Group 2 (p = 0.17). In Group 1 there were 70.4% of males while in Group 2 there were 59.7% of males (p = 0.31). Mean TIT was 4.37±1.35 hours in Group 1 and 9.03±1.59 hours in Group 2 (p < 0.0001). Group 1 had a mean GFR of 47.97±10.23 ml/min/1.73 m^2^, while Group 2 had a mean GFR of 48.49±10.62 ml/min/1.73 m^2^ (p = 0.79). A history of hypertension was present in 64.8% of Group 1 participants and 71% of Group 2 participants (p = 0.48) while a history of diabetes was present in 22.2% of Group 1 participants and 22.5% of Group 2 participants (p = 0.96). Prior myocardial infarction was reported by 9.3% in Group 1 and 12.9% in Group 2 (p = 0.53). 38.9% of Group 1 and 53.2% of Group 2 had anterior STEMI, respectively (p = 0.18) (Table 1). The proportion of patients with multivessel in Group 1 was 44.4% and in Group 2 was 48.4% (p = 0.67). The mean LV EF in Group 1 was 39.9±9.8% and in Group 2 was 38.5±7.25% (p = 0.36). When compared to Group 2, Group 1 had a considerably lower incidence of pulmonary edema and cardiogenic shock: in Group 1 and Group 2 3.7% (2 of 54 patients) and 16.1% (10 of 62 patients) (p = 0.034), respectively. In Group 1 and Group 2 ID was 1.9% and 4.8% (p = 0.62) respectively (Table 3).

**Table 3:** Effect of TIT on in-hospital complications. TIT: total ischemic time

Outcome variable	Total n = 116	Group 1 n = 54	Group 2 n = 62	p-value
Pulmonary edema and cardiogenic shock	12 (10.3%)	2 (3.7%)	10 (16.1%)	0.034
In-hospital death	4 (3.4%)	1 (1.9%)	3 (4.8%)	0.62

## Discussion

Numerous studies have demonstrated the connection between TIT and the prognosis following PCI in patients with STEMI [16,22]. This study's key conclusion was the identification of a direct link between TIT and various in-hospital complications, such as pulmonary edema and cardiogenic shock. Regardless of the underlying etiology, chronic kidney disease (CKD) is defined as kidney damage or a GFR of less than 60 ml/min/1.73 m^2^ for three months or more [23]. None of the participants in the current study had CKD before admission. Age-related declines in kidney function are well-expressed by measuring GFR [24,25] and that is why this study used the term “renal dysfunction” rather than “chronic kidney disease.” The TIT value in the current study is significant: the mean TIT in Group 1 was 4.37±1.35, while in Group 2 it was 9.03±1.59 (p < 0.0001). The authors reasoned that there must be statistically significant variations in intra-hospital complications across patients with various TITs if all other data were comparable. In-hospital complications varied between STEMI patients who had renal failure and various TITs, according to the results of the current study. This means that a rise in the TIT, in the presence of RD, affects the course of the disease and results in in-hospital complications. Our study found that patients with TIT higher than 6 hours were more likely to develop complications such as pulmonary edema or cardiogenic shock. Noteworthy is the fact that there is insufficient data in the literature that would indicate the importance of TIT, especially in patients with RD. This study suggests that TIT value deserves more attention in STEMI patients with RD to make in-hospital complications more predictable and may catalyze for other researchers to pay closer attention to TIT values to investigate TIT-related in-hospital complications in patients with RD.

Study limitations

There are some limitations to this study. The study's main limitations are its single-center design and small sample size. The second limitation is that RD was diagnosed solely based on GFR calculations at the time of admission, with no prior documentation.

## Conclusions

TIT is an independent predictor of in-hospital complications. This fact is especially important in patients with concomitant pathologies, such as RD. Our study showed that in STEMI patients with RD, a TIT greater than 6 hours increases the rate of pulmonary edema and cardiogenic shock. These complications worsen the patient's condition and in severe cases can lead to the death of the patient. In STEMI patients with RD, assessing TIT may help predict in-hospital complications.

## References

[1] Kuller LH (2003). Hormone replacement therapy and risk of cardiovascular disease: implications of the results of the Women's Health Initiative. Arterioscler Thromb Vasc Biol.

[2] Di Carli MF, Janisse J, Grunberger G, Ager J (2003). Role of chronic hyperglycemia in the pathogenesis of coronary microvascular dysfunction in diabetes. J Am Col Cardiol.

[3] Wison S, Foo K, Cunningham J (2003). Renal function and risk stratification in acute coronary syndromes. Am J Cardiol.

[4] Herzog CA, Ma JZ, Collins AJ (1998). Poor long-term survival after acute myocardial infarction among patients on long-term dialysis. N Engl J Med.

[5] Gibson CM, Pinto DS, Murphy SA (2003). Association of creatinine and creatinine clearance on presentation in acute myocardial infarction with subsequent mortality. J Am Coll Cardiol.

[6] Luft FC (2000). Renal disease as a risk factor for cardiovascular disease. Basic Res Cardiol.

[7] Gansevoort RT, Correa-Rotter R, Hemmelgarn BR (2013). Chronic kidney disease and cardiovascular risk: epidemiology, mechanisms, and prevention. Lancet.

[8] Sarnak MJ, Levey AS (2000). Cardiovascular disease and chronic renal disease: a new paradigm. Am J Kidney Dis.

[9] Hamm CW, Bassand JP, Agewall S (2012). ESC guidelines for the management of acute coronary syndromes in patients presenting without persistent ST-segment elevation. The Task Force for the management of acute coronary syndromes (ACS) in patients presenting without persistent ST-segment elevation of the European Society of Cardiology (ESC) [Article in Italian]. G Ital Cardiol (Rome).

[10] Van de Werf F, Bax J, Betriu A (2008). Management of acute myocardial infarction in patients presenting with persistent ST-segment elevation: the Task Force on the management of ST-segment elevation acute myocardial infarction of the European Society of Cardiology. Eur Heart J.

[11] Chiamvimonvat V, Goodman SG, Langer A, Barr A, Freeman MR (2001). Prognostic value of dipyridamole SPECT imaging in low-risk patients after myocardial infarction. J Nucl Cardiol.

[12] Schächinger V, Kasper W, Zeiher AM (1996). Adjunctive intracoronary urokinase therapy during percutaneous transluminal coronary angioplasty. Am J Cardiol.

[13] Solhpour A, Chang KW, Arain SA (2016). Ischemic time is a better predictor than door-to-balloon time for mortality and infarct size in ST-elevation myocardial infarction. Catheter Cardiovasc Interv.

[14] Song JX, Zhu L, Lee CY, Ren H, Cao CF, Chen H (2016). Total ischemic time and outcomes for patients with ST-elevation myocardial infarction: does time of admission make a difference?. J Geriatr Cardiol.

[15] Prasad A, Gersh BJ, Mehran R (2015). Effect of ischemia duration and door-to-balloon time on myocardial perfusion in ST-segment elevation myocardial infarction: an analysis from Horizons-Ami trial (harmonizing outcomes with revascularization and stents in acute myocardial infarction). JACC Cardiovasc Interv.

[16] Gao N, Qi X, Dang Y (2022). Association between total ischemic time and in-hospital mortality after emergency PCI in patients with acute ST-segment elevation myocardial infarction: a retrospective study. BMC Cardiovasc Disord.

[17] Kurmi P, Tripathi VD, Tripathi SK (2022). Impact of total ischemic time on clinical outcomes in patients with ST-elevation myocardial infarction: lost time is never found again. Cureus.

[18] Lubovich A, Radzishevsky E, Goldenberg I, Matezky S, Rosenschein U (2018). Total ischemic time and short, intermediate and long term mortality of patients with STEMI treated by primary percutaneous coronary intervention: analysis of data from 2004-2013 ACSIS registry. J Integr Cardiol.

[19] Levey AS, Bosch JP, Lewis JB, Greene T, Rogers N, Roth D (1999). A more accurate method to estimate glomerular filtration rate from serum creatinine: a new prediction equation. Modification of Diet in Renal Disease Study Group. Ann Intern Med.

[20] Ibanez B, James S, Agewall S (2018). 2017 ESC Guidelines for the management of acute myocardial infarction in patients presenting with ST-segment elevation: The Task Force for the management of acute myocardial infarction in patients presenting with ST-segment elevation of the European Society of Cardiology (ESC). Eur Heart J.

[21] Killip T, Kimball JT (1967). Treatment of myocardial infarction in a coronary care unit: a two year experience with 250 patients. Am Coll Cardiol.

[22] Khowaja S, Ahmed S, Kumar R (2021). Time to think beyond door to balloon time: significance of total ischemic time in STEMI. Egypt Heart J.

[23] Levey AS, Eckardt KU, Tsukamoto Y (2005). Definition and classification of chronic kidney disease: a position statement from Kidney Disease Improving Global Outcomes (KDIGO). Kidney Int.

[24] Denic A, Glassock RJ, Rule AD (2016). Structural and functional changes with the aging kidney. Adv Chronic Kidney Dis.

[25] Petrosyan H, Hayrapetyan H, Torozyan S, Tsaturyan A, Buniatyan V, Sedrakyan S (2022). The influence of renal function on in-hospital complications in patients with ST-elevation myocardial infarction. Rom J Cardiol.

